# Supplementary material for: SARS-CoV-2 spike opening dynamics and energetics reveal the individual roles of glycans and their collective impact
Source: Commun Biol. 2022 Nov 3;5:1170. doi: 10.1038/s42003-022-04138-6 (PMC9631587; doi:10.1038/s42003-022-04138-6)
Supplement: Supplementary file 1 — Supplementary Information [file 42003_2022_4138_MOESM1_ESM.pdf]

Supplementary Information for SARS-CoV-2 spike opening dynamics and energetics reveal the individual roles of glycans and their collective impact

Yui Tik Pang[1,†], Atanu Acharya[1,2,†], Diane L. Lynch[1], Anna Pavlova[1], and James C. Gumbart[1,*]

[1]School of Physics, Georgia Institute of Technology, Atlanta, GA 30332, USA
[*]gumbart@physics.gatech.edu
[†]These authors contributed equally to this work.
[2]Current address: BioInspired Syracuse and Department of Chemistry, Syracuse University, Syracuse, NY 13244, USA

Supplementary Note 1

Minimum energy path (MEP). The MEPs were computed using the algorithm by Ensing et al.[1] and the code implemented by Mahmoud Moradi[2]. The two energy minima and the saddle point (the point with lowest energy to cross an energy barrier) were found using a separate in-house python script, and the MEP from the saddle point to each energy minimum was found using the code by Moradi. The paths were smoothed by the least-square fitting B-spline function from python library SciPy[3].

Mean first passage time (MFPT). Let $\lambda(\mathbf{d},\theta)$ be the one-dimensional path parameter that describes the position of the system along the MEP. Assuming the dynamics along λ can be effectively described by a diffusive model, we may apply the Smoluchowski diffusion equation to describe the process[4,5]:

$$\frac{\partial}{\partial t}p(\lambda,t|\lambda_0,0) = \frac{\partial}{\partial\lambda}D(\lambda)e^{-\beta F(\lambda)}\frac{\partial}{\partial\lambda}\left(e^{\beta F(\lambda)}p(\lambda,t|\lambda_0,0)\right) \tag{S1}$$

where $p(\lambda,t|\lambda_0,0)$ is the probability of finding the system at λ after time t, given it was at λ_0 at time 0, $D(\lambda)$ is a position-dependent diffusion constant, and $F(\lambda)$ is the free energy at λ. By rearranging the terms, the MFPT $\bar{\tau}_{FP}$, or the rate inverse k^{-1}, from the initial (A) to the final (B) state is given by:

$$k^{-1} = \bar{\tau}_{FP} = \int_{\lambda_A}^{\lambda_B}\frac{1}{D(\lambda)}e^{\beta F(\lambda)}\int_{\lambda_0}^{\lambda}e^{-\beta F(\lambda')}\,\mathrm{d}\lambda'\,\mathrm{d}\lambda \tag{S2}$$

While $F(\lambda)$ was readily available from the PMF obtained through REUS, $D(\lambda)$ was approximated using a generalized-Langevin-equation-based method, derived by Roux and co-workers and implemented by Gaalswyk et al.[6]. From a time series of a coordinate x_k with the system simulated under a harmonic restraint, the method computes the diffusion constant D_k along x_k by relating it to its velocity autocorrelation function (VACF). With a series of coordinate transformations[7], D_k is transformed from the Cartesian space to the collective variable space (D_{ij}), and then to the path variable space ($D(\lambda)$), which was then inserted back into Eq. S2.

$$D_{ij} = \sum_k D_k\left\langle\frac{\partial z_i}{\partial x_k}\frac{\partial z_j}{\partial x_k}\right\rangle, \quad D(\lambda) = \sum_{ij}D_{ij}\frac{\partial\lambda}{\partial z_i}\frac{\partial\lambda}{\partial z_j} \tag{S3}$$

To determine D_k for each atom and each of its coordinates involved in the definition of the collective variables d and ϕ, we ran a 1-ns simulation for each window along the MEP, with all C_α atoms of the protein restrained by a

force constant of 5 kcal mol^{-1}Å^{-2}. A 2-fs time step was used without the application of HMR. Other simulation parameters were identical to the REUS simulations.

Kinetics analysis. We modeled the down-to-up transition and subsequent binding of the RBD to ACE2 according to the chemical equation

$$\mathrm{D} + \mathrm{ACE2} \underset{k_{\mathrm{close}}}{\overset{k_{\mathrm{open}}}{\rightleftharpoons}} \mathrm{U} + \mathrm{ACE2} \underset{k_{\mathrm{off}}}{\overset{k_{\mathrm{on}}}{\rightleftharpoons}} \mathrm{U{:}ACE2}$$

The associated master equation is

$$\frac{\mathrm{d[D]}}{\mathrm{d}t} = -k_{\mathrm{open}}[\mathrm{D}] + k_{\mathrm{close}}[\mathrm{U}] \tag{S4}$$

$$\frac{\mathrm{d[ACE2]}}{\mathrm{d}t} = k_{\mathrm{off}}[\mathrm{U{:}ACE2}] - k_{\mathrm{on}}[\mathrm{U}][\mathrm{ACE2}] \tag{S5}$$

$$\frac{\mathrm{d[U]}}{\mathrm{d}t} = k_{\mathrm{open}}[\mathrm{D}] - k_{\mathrm{close}}[\mathrm{U}] - k_{\mathrm{on}}[\mathrm{U}][\mathrm{ACE2}] + k_{\mathrm{off}}[\mathrm{U{:}ACE2}] \tag{S6}$$

$$\frac{\mathrm{d[U{:}ACE2]}}{\mathrm{d}t} = -k_{\mathrm{off}}[\mathrm{U{:}ACE2}] + k_{\mathrm{on}}[\mathrm{U}][\mathrm{ACE2}] \tag{S7}$$

The rates k_{open} and k_{close} come from our own calculations. For the glycosylated spike, $k_{\mathrm{open}} = 0.68$/s and $k_{\mathrm{close}} = 1.17 \times 10^3$/s, while for the un-glycosylated spike, $k_{\mathrm{open}} = 7.01 \times 10^3$/s and $k_{\mathrm{close}} = 2.01 \times 10^3$/s. The rates $k_{\mathrm{on}} = 1.40 \times 10^6$/M·s and $k_{\mathrm{off}} = 6.54 \times 10^{-3}$/s were taken from Lan et al.[8] for the RBD alone in order to isolate the effects of binding from conformational changes in the spike. The system of first-order ordinary differential equations was numerically solved up to 250 s, for which all systems reached steady-state populations, using Mathematica along with the initial conditions $[\mathrm{D}]_i$ = 1 nM, $[\mathrm{U}]_i$ = 0 nM, $[\mathrm{U{:}ACE2}]_i$ = 0 nM, and $[\mathrm{ACE2}]_i$ = 15 nM. The concentration chosen for ACE2 is the same order of magnitude as $K_{\mathrm{D}} = 4.67$ nM, and we note that the qualitative results did not change for different values of $[\mathrm{ACE2}]_i$ although absolute populations did shift.

Hydrogen bond analysis. The number of hydrogen bonds formed between RBD-A and other domains of the spike in the REUS trajectories were measured using the HBonds Plugin of VMD. When the distance between a hydrogen-bond donor atom (D) and an acceptor atom (A) is below 3.5 Å and the angle D-H-A is less than 35° from 180°, a hydrogen bond was considered to be formed between D and A. The d-ϕ space is broken into small bins and the number of hydrogen bonds was averaged over each bin.

Contact analysis. Contact calculations were performed using a cutoff distance of 3.5 Å. In other words, when two heavy atoms from two different selections come within 3.5 Å, we count that as one contact. For the analysis done in Figs. 4c-d and S8, the d-ϕ space is broken into small bins and the number of contacts was averaged over each bin. For the analysis done in Fig. S9, we separated conformations from the MEP in two categories and the number of contacts were averaged separately for each category. The two categories were defined by the d values. The conformations with $d \leq 54.9$ Å were considered as the down state, while the rest were taken as the up state.

Antibody accessible surface area (AbASA). AbASA calculations were performed using the solvent-accessible surface area measurement tool as implemented in VMD with a 7-Å probe for each frame along the MEP. For the glycosylated system, we performed two accessible surface area calculations for each frame around each epitope using the *-restrict* option: AbASA of (calculation #1) protein and (calculation #2) protein + glycans. The difference in the accessible surface area obtained from these two calculations gives the coverage provided by the glycans only.

Supplementary Movie 1. The MEP for the spike with (left) and without (right) glycans.

Supplementary Movie 2. The motion of key glycans along the MEP with the glycans rendered red (N122), green (N165), yellow (N234), and orange (N343).

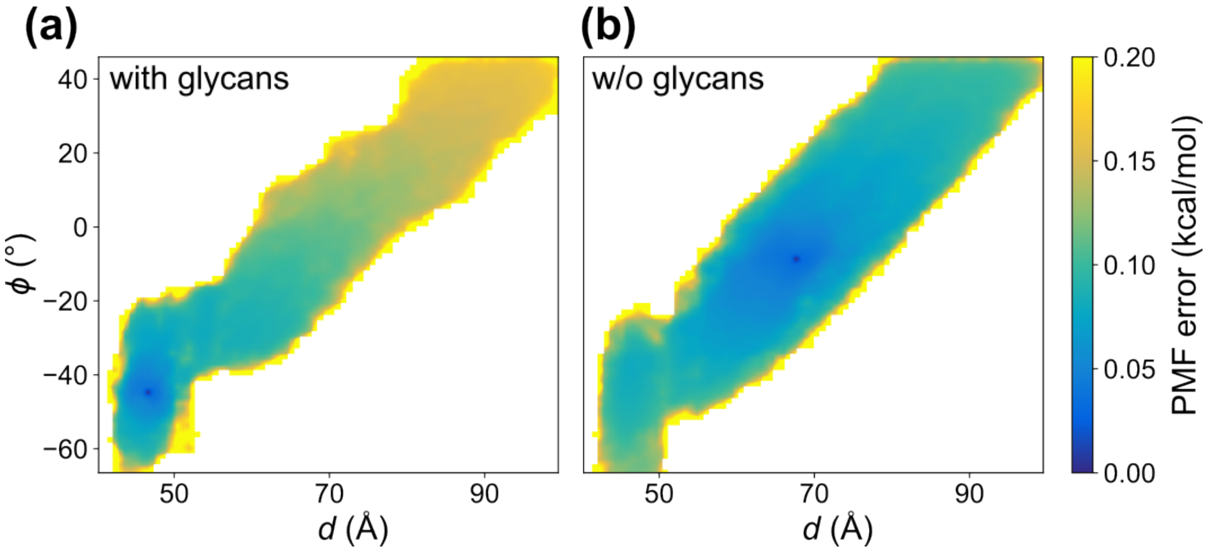

Figure S1. Uncertainties of the free energy differences with the lowest point on the PMF computed by MBAR for the (a) glycosylated and (b) un-glycosylated system.

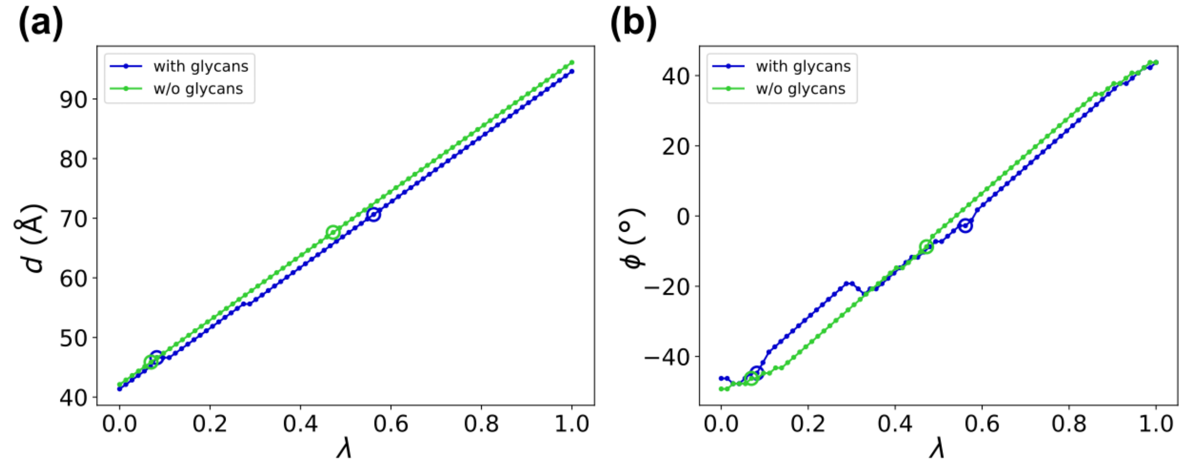

Figure S2. Path parameter λ. (a,b) The path parameter is defined as a value going from zero to one along the MEP from the bottom-left to the top-right corner of the PMF. The corresponding d and ϕ are plotted against λ in (a) and (b), respectively. The position of the down- and up-state energy minima are marked as empty circles.

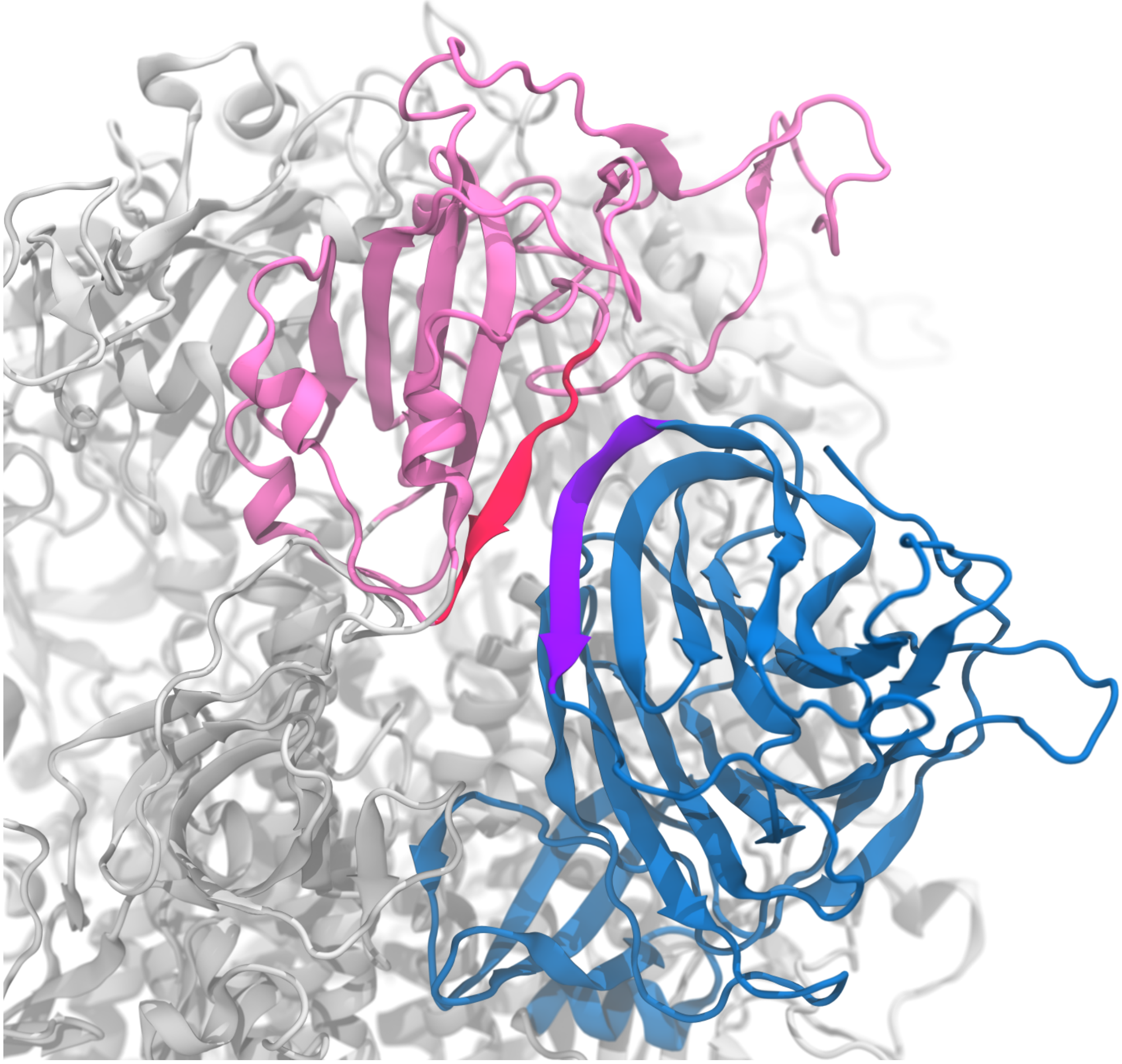

Figure S3. Snapshot from the un-glycosylated REUS simulation showing the β-sheets of RBD-A (pink) and NTD-B (blue) align with each other and form strong hydrogen bonds when the RBD-A is in the up state.

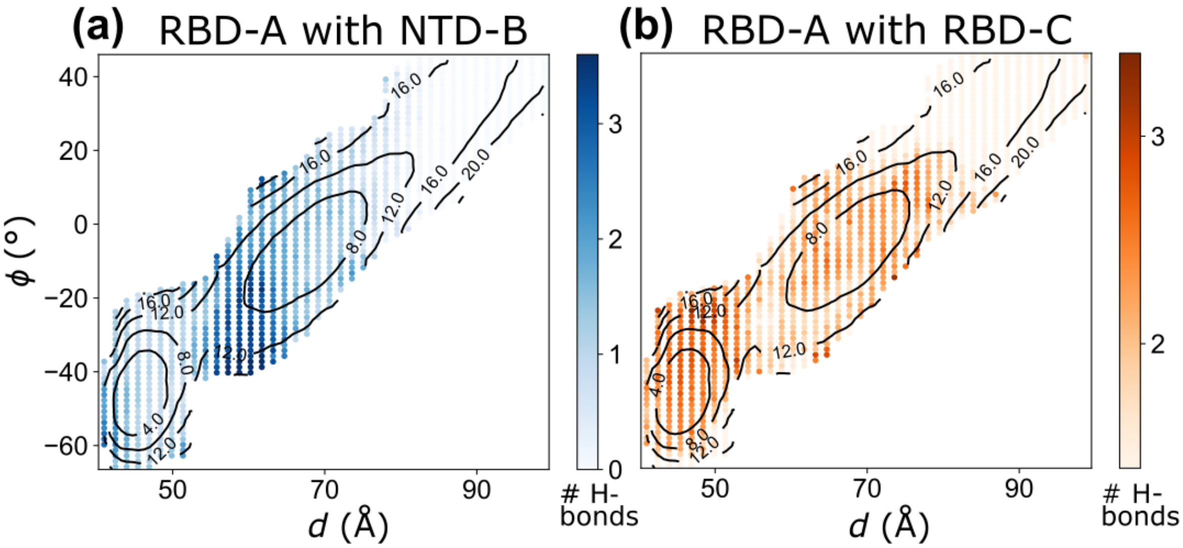

Figure S4. Hydrogen bond analysis for the glycosylated system. The average number of hydrogen bonds formed (a) between RBD-A and NTD-B and (b) between RBD-A and RBD-C are plotted against the two collective variables, d and ϕ. Contour lines of the PMF of the glycosylated system are plotted on top to show the location of the energy barrier.

	glycosylated			un-glycosylated		
Domains	x (Å)	y (Å)	z (Å)	x (Å)	y (Å)	z (Å)
SD1-B	-31.6 ± 1.1	14.1 ± 1.1	-13.1 ± 1.0	-31.4 ± 0.8	14.7 ± 1.2	-12.4 ± 1.0
SD1-A	2.2 ± 1.4	-34.2 ± 1.1	-11.8 ± 1.6	2.6 ± 1.7	-34.8 ± 1.2	-10.9 ± 2.1
SD2-A	26.5 ± 1.4	-25.4 ± 1.6	11.6 ± 1.2	25.6 ± 1.2	-25.3 ± 1.5	12.5 ± 1.0
NTD-A	45.8 ± 1.7	-11.0 ± 1.5	-21.8 ± 0.9	45.2 ± 1.6	-11.2 ± 1.6	-21.7 ± 1.1

Table S1. The average position of the stationary domains used to define the two collective variables throughout the REUS simulations and their standard deviations.

	PMF cutoff (kcal/mol)					Number of windows				
Round	1	2	3		4	1	2	3		4
			Down	Up				Down	Up	
WT with glycans	30	28	25	26	∞	387	422	178	700	1049
WT w/o glycans	24	12	25	25	∞	392	356	257	669	1211
diproline mutant w/o glycans	28	–	–	–	–	353	–	–	–	–

Table S2. The PMF cutoffs for a window to be retained for the next stage of REUS simulation and the resulting number of REUS windows.

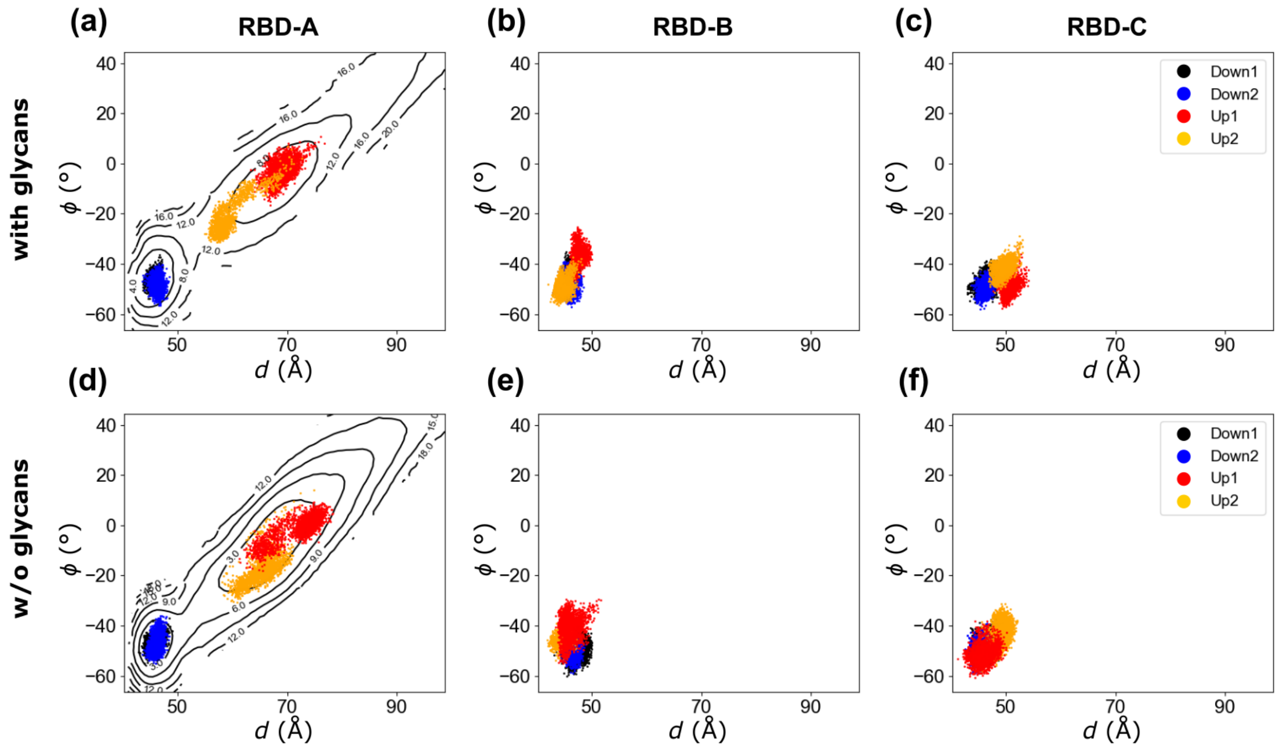

Figure S5. Collective variable d vs. ϕ for the RBDs in 2×2-µs equilibrium simulations. (a-c) Glycosylated system. (d-f) Un-glycosylated system. The S-protein protomers in the down state, including (a,d) RBD-A that started at the down state (black/blue), (b,e) RBD-B, and (c,f) RBD-C, all remained in the free energy well of the down state for all replicas, with or without glycans. The up-state RBD-A (red/yellow) was less stable in comparison.

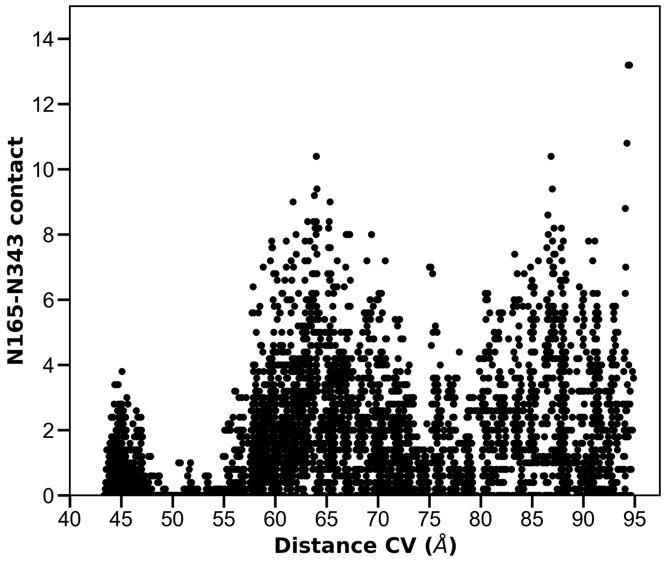

Figure S6. Contact between glycans at N165 and N343 along the MEP.

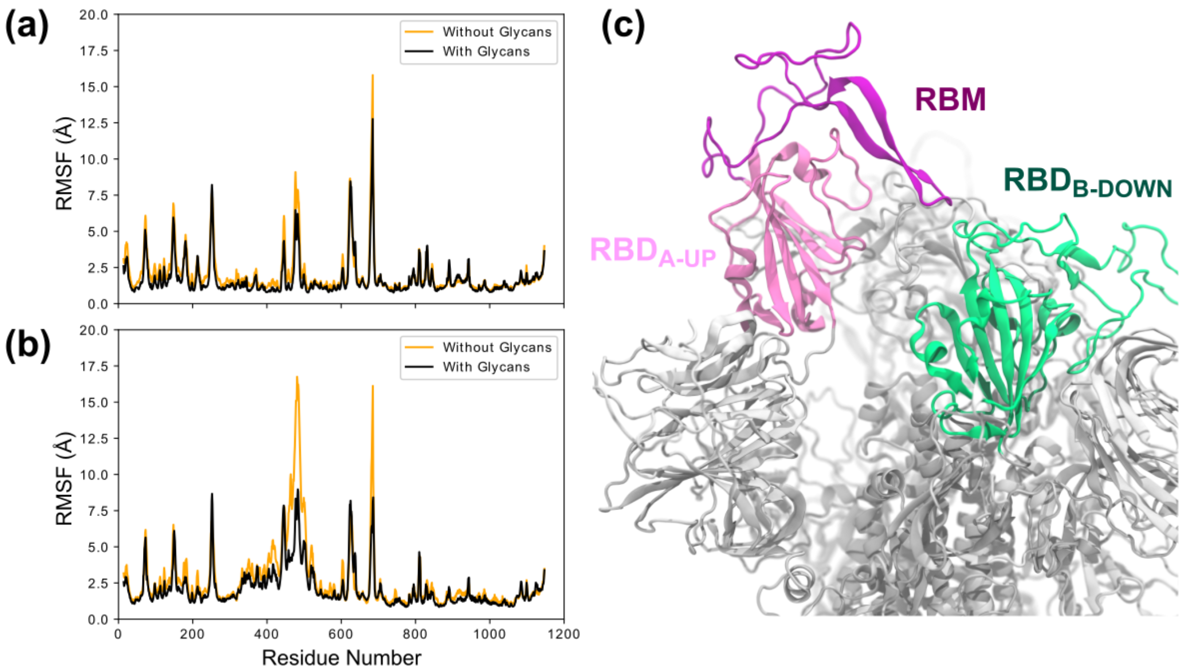

Figure S7. Root-mean-square fluctuation (RMSF) for equilibrium simulations. (a) RMSF of the down protomers, averaged over the 3 protomers and 2 replicas. (b) RMSF of the single up protomer, averaged over both replicas. RMSF computed after alignment and averaging over all C_α atoms of the trimer. (c) Snapshot from the equilibrium simulation without glycans, showing the RBM region (438–506) of the up-state protomer attached to the neighboring RBD-B.

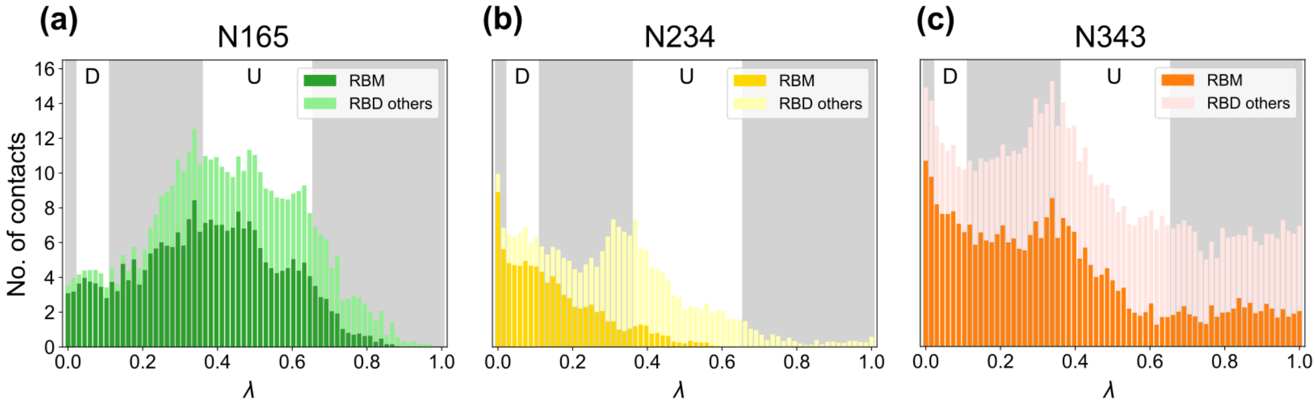

Figure S8. The number of contacts along the MEP between the glycans at (a) N165, (b) N234 and (c) N343, and RBD-A, which is separated into the RBM (439–506) and the non-RBM.

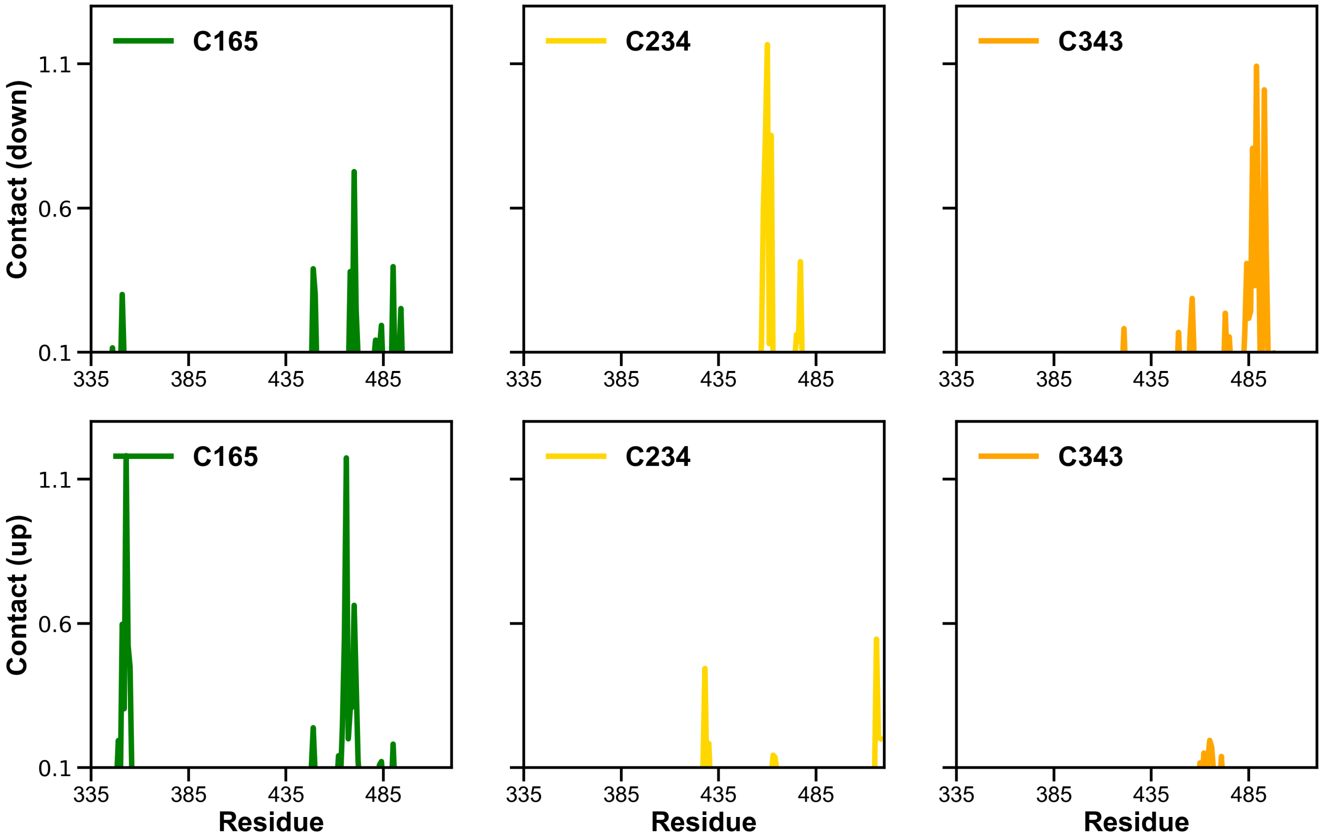

Figure S9. Average contact between glycans and RBD residues in up and down conformations of the MEP. Conformations in the range 43.1 Å ≤ d ≤ 48.9 Å were considered as the down state, while the conformations with 60.1 Å ≤ d ≤ 75.6 Å were taken as the up state.

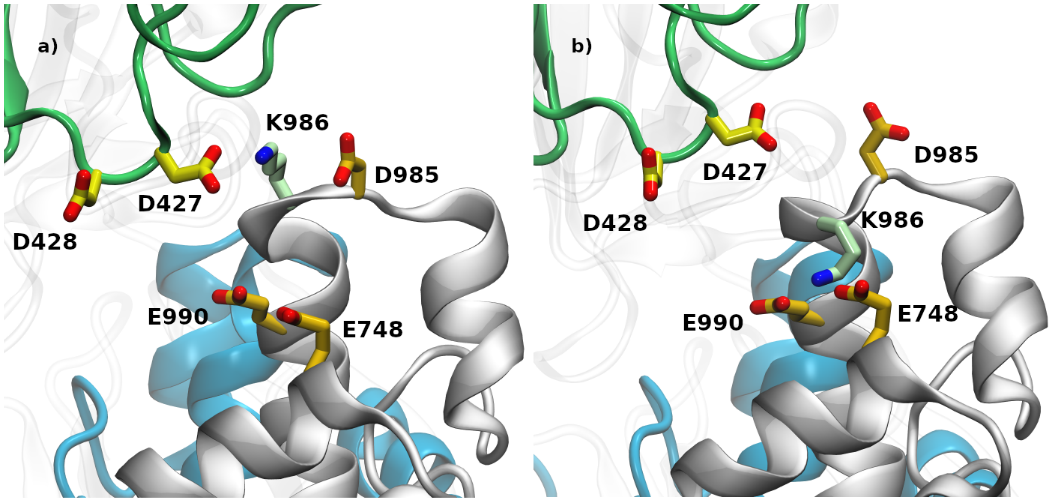

Figure S10. Intra-protomer and inter-protomer K986 interactions. K986 transiently interacts with a) D427 from a neighboring protomer or b) D985, E748, and E990 from the same one. D427, D428, and the green ribbon are from protomer B while K986, D985, E748, E990, as well as the silver and blue ribbons, are from protomer A.

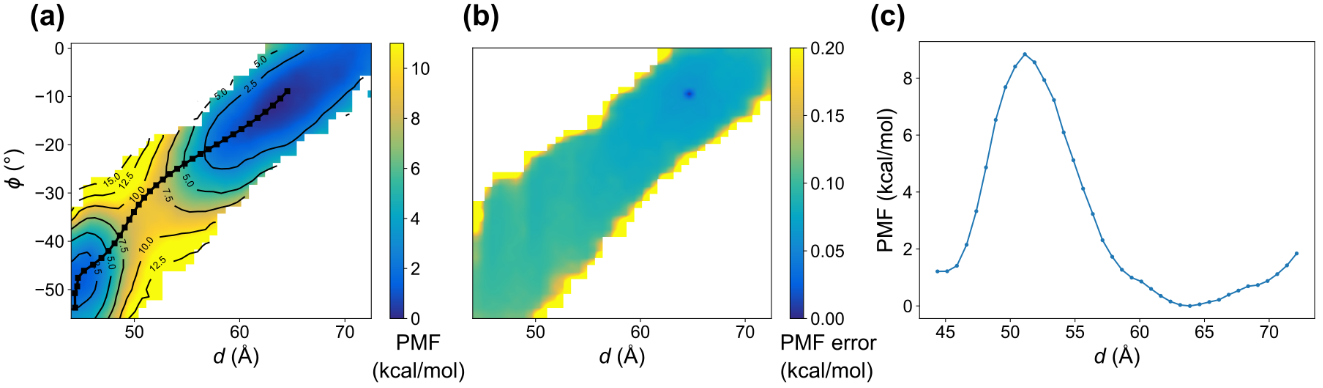

Figure S11. PMF of the diproline-mutated un-glycosylated spike. (a) The 2D PMF along two collective variables, d and ϕ. The black lines show the MEP. (b) Uncertainties of the free energy differences with the lowest point on the PMF computed by MBAR. (c) The free energies are projected onto d and plotted as an 1D PMF.

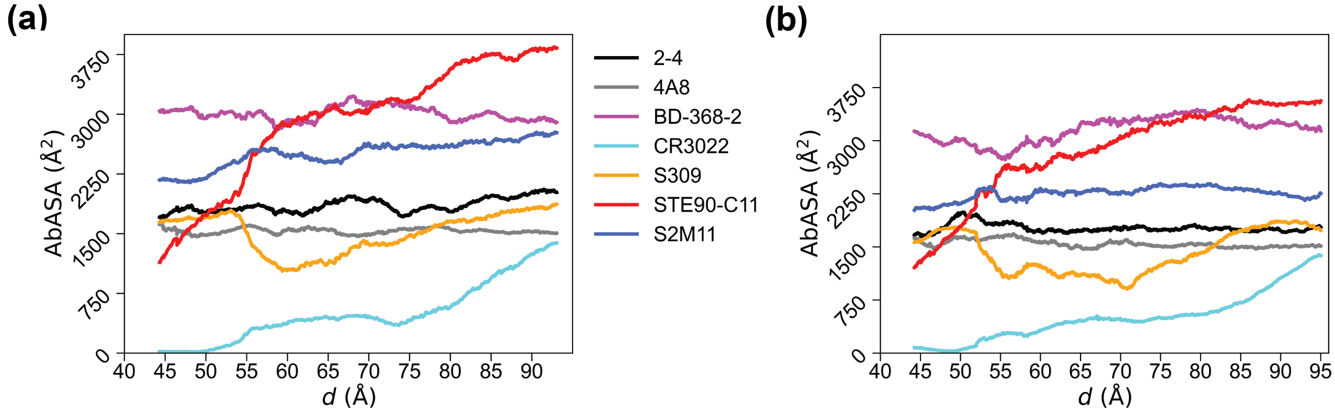

Figure S12. Epitope analysis of selected antibodies. Exposed area on antibody epitopes without glycans along the MEP obtained (a) with glycans and (b) without glycans. All accessible surface area calculations were performed using a 7-Å probe.

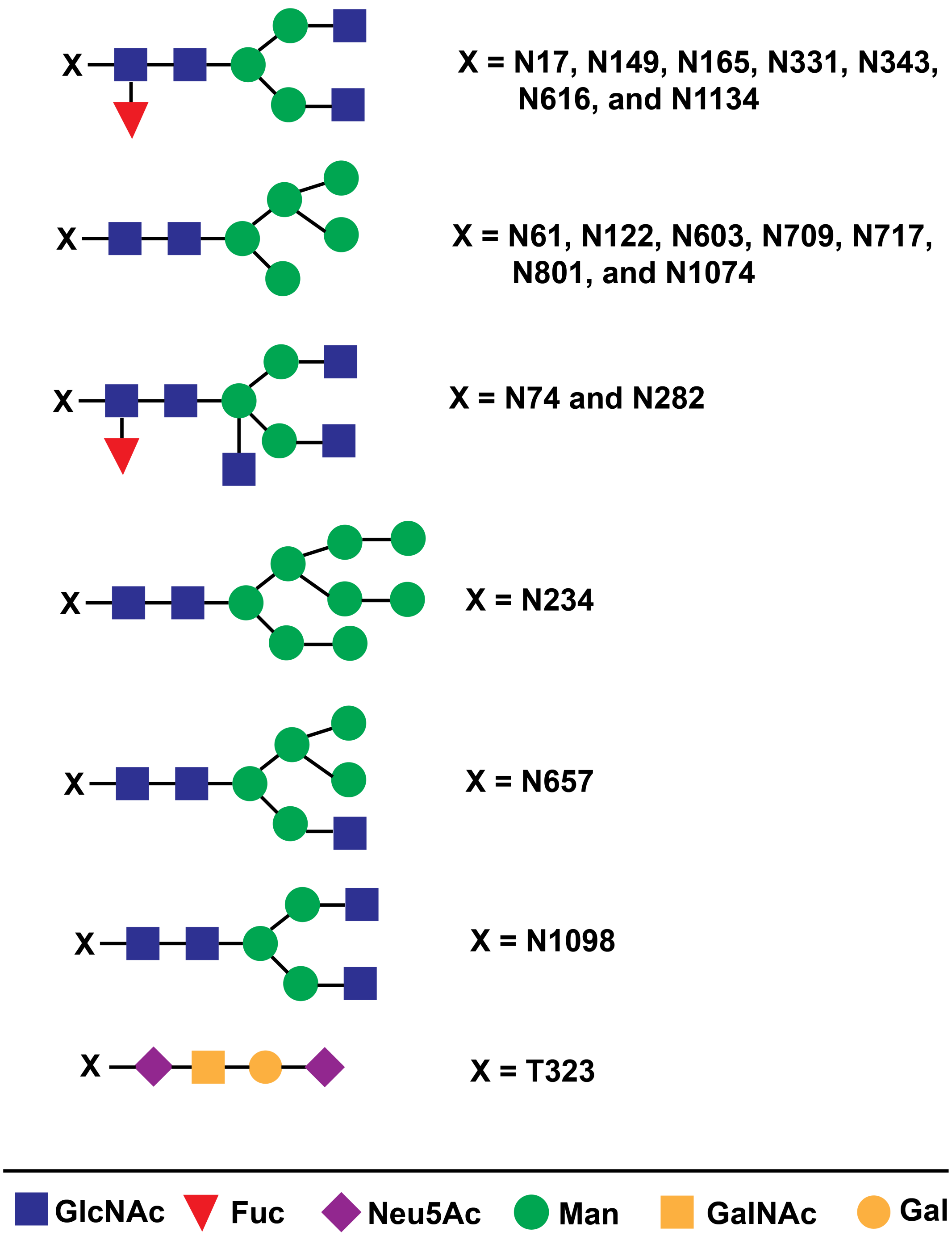

Figure S13. Glycan compositions used in this study.

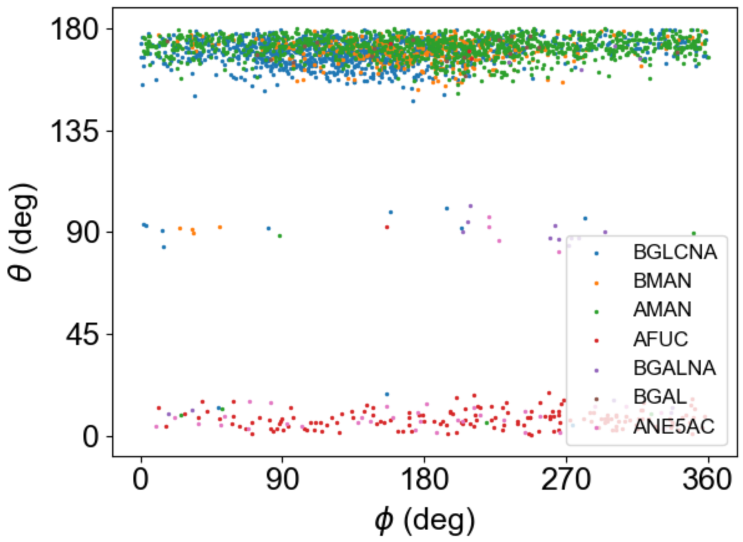

Figure S14. Cremer-Pople angles[9] of the glycans after simulated annealing.

Table S3. Details of the antibodies investigated in this study. Antibodies 2-4[10], 4A8[11], and BD-368-2[12] were obtained from B cells of patients after SARS-CoV-2 infection, while S309[13] was extracted from B cells of SARS-CoV infected patients. Antibodies CR3022[14] and STE90-C11[15] were created from phage libraries.

Antibody	Binds to	Residues in epitope	PDB id	Reference
2-4	SARS-CoV-2 RBD	446–447, 449, 452–453, 455–456, 483–487, 489, 490, 492–496, 498	6XEY	Liu et al.[10]
4A8	SARS-CoV-2 NTD	143–148, 150–152, 158, 245–251, 256–257	7C2L	Chi et al.[11]
BD-368-2	SARS-CoV-2 RBD	346, 351, 444–447, 449–450, 452, 470, 472, 478–486, 490, 492, 494, 498	7CHH	Du et al.[12]
CR3022	SARS-CoV-2 RBD	369–372, 374–386, 389–390, 392, 427–430, 515–517, 519	6W41	Yuan et al.[14]
S309	SARS-CoV/SARS-CoV-2 RBDs	333–335, 337, 339–341, 343–346, 354, 356–361, 440, 441, 444, 509	6WPS	Pinto et al.[13]
STE90-C11	SARS-CoV-2 RBD	403, 405, 406, 408, 409, 415–417, 420, 421, 449, 453, 455–460, 473–477, 486–487, 489, 493–496, 498, 500–505	7B3O	Bertoglio et al.[15]
S2M11	SARS-CoV-2 RBD	Up protomer: 339, 342, 343, 345, 367, 368, 371–374, 436, 440, 441, 444, 339, 342, 343, 345, 367, 368, 371–374, 436, 440, 441, 444<br>Down protomer: 446, 447, 449, 452, 455, 456, 483–490, 492–494, 496, 498	7K43	Tortorici et al.[16]

Supplementary References

1. Ensing, B., Laio, A., Parrinello, M. & Klein, M. L. A recipe for the computation of the free energy barrier and the lowest free energy path of concerted reactions. *J. Phys. Chem. B* **109**, 6676–6687 (2005).
2. Moradi, M., Babin, V., Roland, C., Darden, T. A. & Sagui, C. Conformations and free energy landscapes of polyproline peptides. *Proc. Natl. Acad. Sci. U. S. A.* **106**, 20746–20751 (2009).
3. Virtanen, P. *et al.* SciPy 1.0: Fundamental Algorithms for Scientific Computing in Python. *Nat. Methods* **17**, 261–272 (2020).
4. Smoluchowski, M. V. Über Brownsche Molekularbewegung unter Einwirkung äußerer Kräfte und deren Zusammenhang mit der verallgemeinerten Diffusionsgleichung. *Ann. Phys.* **353**, 1103–1112 (1916).
5. Singharoy, A., Chipot, C., Moradi, M. & Schulten, K. Chemomechanical Coupling in Hexameric Protein-Protein Interfaces Harnesses Energy within V-Type ATPases. *J Am Chem Soc* **139**, 293–310 (2017).
6. Gaalswyk, K., Awoonor-Williams, E. & Rowley, C. N. Generalized Langevin Methods for Calculating Transmembrane Diffusivity. *J. Chem. Theory Comput.* **12**, 5609–5619 (2016).
7. Fakharzadeh, A. & Moradi, M. Effective Riemannian Diffusion Model for Conformational Dynamics of Biomolecular Systems. *J. Phys. Chem. Lett.* **7**, 4980–4987 (2016).
8. Lan, J. *et al.* Structure of the SARS-CoV-2 spike receptor-binding domain bound to the ACE2 receptor. *Nature* **581**, 215–220 (2020).
9. Cremer, D. & Pople, J. A. General definition of ring puckering coordinates. *J. Am. Chem. Soc.* **97**, 1354–1358 (1975).
10. Liu, L. *et al.* Potent neutralizing antibodies against multiple epitopes on SARS-CoV-2 spike. *Nature* **584**, 450–456 (2020).
11. Chi, X. *et al.* A neutralizing human antibody binds to the N-terminal domain of the Spike protein of SARS-CoV-2. *Science* **369**, 650–655 (2020).
12. Du, S. *et al.* Structurally Resolved SARS-CoV-2 Antibody Shows High Efficacy in Severely Infected Hamsters and Provides a Potent Cocktail Pairing Strategy. *Cell* **183**, 1013–1023 (2020).
13. Pinto, D. *et al.* Cross-neutralization of SARS-CoV-2 by a human monoclonal SARS-CoV antibody. *Nature* **583**, 290–295 (2020).
14. Yuan, M. *et al.* A highly conserved cryptic epitope in the receptor binding domains of SARS-CoV-2 and SARS-CoV. *Science* **368**, 630–633 (2020).
15. Bertoglio, F. *et al.* SARS-CoV-2 neutralizing human recombinant antibodies selected from pre-pandemic healthy donors binding at RBD-ACE2 interface. *Nat. Commun.* **12**, 1577 (2021).
16. Tortorici, M. A. *et al.* Ultrapotent human antibodies protect against SARS-CoV-2 challenge via multiple mechanisms. *Science* **370**, 950–957 (2020).
