# Supplementary material for: SARS-CoV-2 spike opening dynamics and energetics reveal the individual roles of glycans and their collective impact
Source: Commun Biol. 2022 Nov 3;5:1170. doi: 10.1038/s42003-022-04138-6 (PMC9631587; doi:10.1038/s42003-022-04138-6)
Supplement: Supplementary file 2 — Description of Additional Supplementary Files [file 42003_2022_4138_MOESM2_ESM.pdf]

Description of Additional Supplementary Files

File name: Supplementary Data 1
Description: The source data behind the PMFs in Figure 2.

File name: Supplementary Movie 1
Description: The MEP for the spike with (left) and without (right) glycans.

File name: Supplementary Movie 2
Description: The motion of key glycans along the MEP with the glycans rendered red (N122), green (N165), yellow (N234), and orange (N343).
